# Ultrastructure of Synaptic Connectivity within Subregions of the Suprachiasmatic Nucleus Revealed by a Genetically Encoded Tag and Serial Blockface Electron Microscopy

**DOI:** 10.1523/ENEURO.0227-23.2023

**Published:** 2023-08-23

**Authors:** Hugo Calligaro, Azarin Shoghi, Xinyue Chen, Keun-Young Kim, Hsin Liu Yu, Brian Khov, Benjamin Finander, Hiep Le, Mark H. Ellisman, Satchidananda Panda

**Affiliations:** 1 Salk Institute for Biological Studies, La Jolla, CA 92037; 2 Department of Neurosciences, University of California at San Diego School of Medicine, La Jolla, CA 92161; 3 National Center for Microscopy and Imaging Research, University of California, San Diego, La Jolla, CA 92161

**Keywords:** connectome, melanopsin, retinal ganglion cells, suprachiasmatic nucleus

## Abstract

The hypothalamic suprachiasmatic nucleus (SCN) is the central circadian pacemaker in vertebrates. The SCN receives photic information exclusively through melanopsin-expressing retinal ganglion cells (mRGCs) to synchronize circadian rhythms with the environmental light cycles. The SCN is composed of two major peptidergic neuron types in the core and shell regions of the SCN. Determining how mRGCs interact with the network of synaptic connections onto and between SCN neurons is key to understand how light regulates the circadian clock and to elucidate the relevant local circuits within the SCN. To map these connections, we used a newly developed Cre-dependent electron microscopy (EM) reporter, APEX2, to label the mitochondria of mRGC axons. Serial blockface scanning electron microscopy was then used to resolve the fine 3D structure of mRGC axons and synaptic boutons in the SCN of a male mouse. The resulting maps reveal patterns of connectomic organization in the core and shell of the SCN. We show that these regions are composed of different neuronal subtypes and differ with regard to the pattern of mRGC input, as the shell receives denser mRGC synaptic input compared with the core. This finding challenges the present view that photic information coming directly from the retina is received primarily by the core region of the SCN.

## Significance Statement

The hypothalamic suprachiasmatic nucleus (SCN) in the vertebrate brain serves as the central pacemaker regulating circadian rhythm throughout the body and as the principal hub for entraining circadian rhythm with the ambient light/dark cycle. Cellular and molecular studies have suggested heterogeneity of the SCN neurons and their connectivity, yet an ultrastructural characterization of the SCN connectivity is still lacking. In order to systematically investigate the connectivity within the SCN, we used a recently developed Cre-dependent electron microscopy (EM) reporter, APEX2, to specifically label mitochondria of melanopsin-expressing retinal ganglion cells (mRGCs), and SBEM to produce image volumes of the two functional subregions of the SCN, the core and the shell. Our findings unveil several differences between the subregions, including synaptic input (retinal and nonretinal) and the density of network of dendrites forming dendro-dendritic synapses. In addition, we established morphologic criteria for discriminating between different types of axonic boutons.

## Introduction

In mammals, the circadian organization of physiology, behavior, and metabolism is necessary for a healthy lifespan. Although circadian rhythms are cell-autonomous and found in almost every cell, the suprachiasmatic nucleus (SCN), which is a brain region above the optic chiasma in the ventral hypothalamus, plays an indispensable role in organizing these rhythms. Surgical ablation of the SCN or targeted disruption of circadian clock genes in the SCN abolishes circadian rhythms in behavior (Moore and Eichler, 1972; Stephan and Zucker, 1972). The neurons of the SCN generate autonomous circadian oscillations and orchestrate the timing of circadian rhythms in other tissues through synaptic and humoral regulation (Welsh et al., 1995, 2010; Abrahamson and Moore, 2001). The SCN also exclusively drives the adaptation of circadian rhythms to seasonal changes in day length through retinal light input to SCN neurons (Coomans et al., 2015). Given the central role of the SCN in the circadian organization and behavioral adaptations to ambient light, there is intense interest in understanding the cellular composition, cytoarchitecture, synaptic organization, and molecular properties of the SCN.

In mice, each half of the SCN is composed of roughly 10,000 neurons and is divided into two main subdivisions, namely the core (ventral) and the shell (dorsal), based on the expression of neuropeptides. VIP-expressing and GRP-expressing cells localize primarily to the core region, whereas AVP-expressing and calretinin-expressing cells are found primarily in the shell region (Moore, 1996; Abrahamson and Moore, 2001; Yan and Silver, 2002; Fernandez et al., 2016). The SCN receives primary photic input from the retina and secondary photic input from the intergeniculate nucleus (IGL) and the raphe nucleus (Abrahamson and Moore, 2001; Hannibal and Fahrenkrug, 2006; Welsh et al., 2010). SCN neurons are believed to make extensive reciprocal connections with each other and to project to other hypothalamic and thalamic brain regions (Welsh et al., 2010). Another structural characteristic of the SCN is the presence of a communication network among neurons that does not depend on axonal communication, but rather another type of synapse, dendro-dendritic chemical synapses (DDCSs). These structures are relatively rare in the mammalian brain and their roles remain unclear. DDCSs are associated with several types of peptidergic neurons in the hypothalamus (Güldner and Wolff, 1974; Theodosis et al., 1981; Theodosis and Poulain, 1984; Alonso et al., 1985), where it is proposed they synchronize neuronal activity (Theodosis et al., 1981). The same function has been proposed in the SCN, particularly for VIP neurons (Bosler and Beaudet, 1985). To our knowledge, the specific cell types in the SCN that communicate via DDCS have not been identified. We have previously shown that dendrites forming DCCS in the SCN are not isolated, but instead form a complex network and these dendrites receive dense synaptic inputs from retinal and nonretinal sources (Kim et al., 2019). However, the importance and distribution of dendrites forming DDCS within the SCN remain unclear.

While great progress has been made in understanding the gene expression patterns, electrophysiological properties, and peptidergic contents of SCN neurons, we still know very little about the nature and extent of connectivity within the SCN. The SCN is composed of densely packed neurons with no apparent laminar or organizational stratification as seen in many other brain regions. Systematic sparse labeling of rat SCN neurons has led to their classification into subtypes based on morphology, specifically the number of primary neurites (van den Pol, 1980). Electron microscopy (EM) analyses of the SCN have also revealed axons of various origins, both from inside (Bosler and Beaudet, 1985; Castel and Morris, 2000) and outside the SCN (Card et al., 1981; Castel et al., 1993; Kim et al., 2019), but their distribution and connectivity pattern remain largely unknown.

The emergence of connectomic EM methods such as serial blockface electron microscopy (SBEM), as well as genetically encoded electron microscopy tags and viral-mediated expression of these tags in specific neurons, provides powerful new approaches for investigating the connectivity patterns of nuclei like the SCN. Recently, we used a genetically encoded light and EM (GEM) tag (miniSOG) to specifically label retinal ganglion cells that express melanopsin [melanopsin-expressing retinal ganglion cells (mRGCs)] and are monosynaptically connected to the SCN and other brain regions (Kim et al., 2019). However, the nature of this GEM tag made it difficult to fully characterize the synaptic connectivity of the labeled cells. In addition, the study was not focused entirely on the SCN. Here, we modified a recently developed tag system (Lam et al., 2014; Ramachandra et al., 2021) to allow viral-mediated expression in Cre-dependent mice, as well as the specific labeling of mitochondria. This strategy can be used with mini-SOG or APEX2 to restrict the marker to mitochondria, leaving other subcellular domains with more native electron density. A similar approach was recently used by Zhang and colleagues (Zhang et al., 2019). This labeling approach now allows us to characterize the synaptic connectivity of mRGCs and all connections within the SCN. Here, we conducted a comparative analysis of the two main subdivisions of the SCN, the core and the shell from the same animal. We asked four questions. What are the properties of SCN cells (density, nuclei volume, number of neurites)? What are the connectivity properties of SCN neurons (somatic and dendritic synapses, balance between retinal and nonretinal input)? What are the characteristics of axons and axonal boutons in the SCN (volume, number of mitochondria, synaptic vesicles, dendritic intrusions, synaptic partners). Finally, can we use morphologic characteristics to identify mRGCs axons without specific labeling?

## Materials and Methods

### Mice

All animal experiments and use were approved by the Salk Institute Institutional Animal Care and Use Committee (IACUC). Six- to eight-month-old C57BL/6J OPN4Cre/+ mice (Hatori et al., 2008) were used for protocol optimization. All mice were housed under 12/12 h light/dark cycles of 100 lux of white light, in a temperature-controlled room with food *ad libitum*. Collection of the SCN was done at Zeitgeber Time 6. Both SCN volumes are from the same mouse, an eight-month-old male.

### Vector construction

A mitochondria-specific sequence was cloned into the 3′ end of the APEX2 construct (Lam et al., 2014; Ramachandra et al., 2021) and was inserted in an inverted orientation between the lox sites in an AAV2-DIO vector (Cardin et al., 2009) to create AAV-DIO-Mito-V5-APEX2 (Addgene plasmid #72480; RRID:Addgene_72480). To enable an extended search of the region of interest (ROI) under a fluorescent microscope, we co-injected the APEX2 virus with a tdTomato-farnesyl virus to the Opn4^Cre/+^ mice. AAV-DIO-Mito-V5-APEX2 and AAV2-DIO-tdTomato-farnesyl were produced by the Salk Gene Transfer, Targeting and Therapeutics Viral Vector Core Facility at titers of 2.0 × 10^11^ TU/ml and 9.4 × 10^11^ TU/ml, respectively.

### Vector injection

Anesthesia was induced with isoflurane (4%) and maintained (2%) until the end of the procedure. The mouse is placed under a dissection microscope so one eye is completely visible. Gentle pressure is applied around the eye so the edge of the sclera is visible. A small incision is made with a 27-gauge insulin needle 0.5 mm posterior to the ora serrata. The vector is loaded into a Hamilton microliter syringe with a 34-gauge beveled needle mounted on a micromanipulator. The micromanipulator is used to insert the loaded needle through the incision. The vector is slowly injected and allowed to diffuse through the vitreous humor for 2 min. The whole procedure is then repeated on the other side with the appropriate vector. After both eyes have been injected, the animal is removed from isoflurane anesthesia and lubricant eye ointment (AKORN) is applied to both eyes. The animal is placed in a clean cage to recover and is returned to its home cage when righting reflex is restored, after 1–2 min.

#### Tissue collection and sample preparation

Four weeks after the injection, the animal was perfused with 4% formaldehyde-0.1% glutaraldehyde in 0.15 m cacodylate buffer. We collected then postfixed the retinas and the brain with 4% formaldehyde in 0.1 m PBS on ice for 2 h. Both retinas were observed in fluorescence microscopy to confirm the homogeneous infection of RGCs on the whole surface of the retina before processing the brain. The brain was cut into 100-µm coronal slices using a vibratome (Leica VT1000S). The SCN was then localized on a Leica SPE II inverted confocal microscope using the tdTomato fluorescence marking mRGC axons. As expected, the SCN was visible on several adjacent sections. We choose a central section, corresponding to −0.46 mm from bregma on Paxinos and Franklin mouse brain atlas (Paxinos and Franklin, 2001), where both regions were clearly visible. The ventromedian (core) and dorsol lateral (shell) regions were each isolated from a different hemisphere.

### SBEM staining and imaging

The brain slice was prepared for SBEM as previously described (Deerinck et al., 2010; Tsang et al., 2018; Kim et al., 2019). To reveal APEX2-expression in mRGC axons, the brain sections was incubated in a diaminobenzidine [DAB (0.05%)]/H_2_O_2_ (0.015%) in 0.1 m sodium cacodylate buffer for 1 h. After the reaction, the brain section was washed with 0.1 m sodium cacodylate buffer. The brain section went through osmium staining for SBEM. Briefly, the brain section was incubated in OsO_4_ (2%)/potassium ferrocyanide (1.5%)/CaCl_2_ (2 mm) in 0.15 m sodium cacodylate for 1 h. Then the brain section was washed in water and transferred to filtered thiocarbohydrazide (0.5%) for 30 min. The brain section was then washed in water and incubated in OsO_4_ (2%) for 30 min. Finally, the brain section was rinsed with water and transferred to filtered aqueous uranyl acetate (2%) overnight. The brain section was then imbedded in resin following standard protocol and mounted for SBEM.

The brain volumes were collected in 2.0- to 2.4-kV accelerating voltages, with a raster size of 20,000 × 20,000 or 24,000 × 24,000 and pixel dwell time of 0.5 −1.5 μs. The pixel size was 7.4 nm, and section thickness was 50 nm. Before each volume was collected, a low-magnification (∼500×) image was collected of the block face to confirm the anatomic location of the volume based on tissue landmarks. Once a volume was collected, the histograms for the slices throughout the volume stack were normalized to correct for drift in image intensity during acquisition. Digital micrograph files were normalized using Digital Micrograph and then converted to MRC format. The stacks were converted to eight-bit, mosaics were stitched, and volumes were manually traced for reconstruction and analysis.

### Three-dimensional reconstruction and analysis

To analyze these datasets, we used the publicly available software package IMOD, specifically developed for the visualization and analysis of EM datasets in three dimensions (Kremer et al., 1996; http://bio3d.colorado.edu/imod/). Cross-sectional contours were manually traced for consecutive data slices in the *z* dimension to determine the boundaries of user-defined objects. For some objects, contours were traced in every other data slice in the *z* dimension. These contour profiles were used for three-dimensional volumetric reconstruction of the cell body, axons, boutons, and organelles.

### Manual segmentation

#### Length and volume quantification

To measure the length of neurites, we fully traced each branch individually by marking the neurite center every five z-steps. To calculate the volume of organelles, we fully segmented them individually every two z-steps. The length and volume values are then extracted using *imodinfo*.

#### Synaptic input quantification

Synaptic sites were identified by scanning the neurites previously skeletonized and finding areas with synaptic vesicles close to the cell membrane and at least one of the following criteria: proximity between the presynaptic and postsynaptic element cell membranes and presence of dendritic intrusions. Synapses are then classified between axodendritic chemical synapse (ADCS) and dendrodendritic chemical synapse (DDCS) by identifying both elements using the specific criteria listed below. For ADCS, the axonic element is classified as either mRGCs or nonretinal based on the APEX2 labeling of mitochondria. To avoid overestimation and underestimation of the density of synapses on dendrites, we excluded from the analysis all dendrites shorter than 2 µm. This criterion excluded three proximal dendrites in the core.

#### Identification of axons and dendrites

To discriminate between axons and dendrites, we look for different characteristic elements. The dendrites have usually a consistent and large diameter, with dendritic spines, dendritic intrusions, and are mostly postsynaptic elements of synapses. The axons have a smaller diameter with swelling forming synapses, receive dendritic intrusions, and are systematically the presynaptic element of synapses.

#### Counting of nuclei, astrocytes, stigmoid bodies, nucleoli

To count the nuclei, astrocytes, stigmoid bodies (SBs), and nucleoli in the two datasets, a five-by-five grid was created. The entirety of the dataset was scanned systematically by starting from one corner and going through all the created grids and marking each of the nuclei, astrocytes, stigmoid bodies, and nucleoli with a different color. This process was done until all the areas in the volume sets of the ventral and dorsal SCN were covered.

#### Bouton identification

For the identification of boutons, we looked for areas of the axon that were swollen to a diameter at least twice as large as the average diameter of the axon. Our criteria for swelling to be considered a synaptic bouton included the presence of at least one synapse identified as synaptic vesicles close to the cell membrane at contact with a postsynaptic element. This postsynaptic element can be a direct contact with a neighboring cell or a dendritic intrusion.

#### Identification of myelinated axons

For the segmentation of myelinated axons, we created a five-by-five grid and started at the lowest z-step in the dataset, and looked for axons with thick dark myelin around them. These axons were traced to obtain an estimate of their length and branching pattern. This process was done until all the areas in the volume sets of the ventral and dorsal SCN were covered.

#### Training and quality control

All person involved in manual tracing were first trained to identify and label specific structures (axons, dendrites, soma) and ultrastructures (mitochondria, synapses, stigmoid bodies, etc.). A particular attention was given to the identification of mitochondria labeled with APEX2. All data were systematically re-checked at least once by a different person than the one who initially produced it.

### Statistical analysis

Results are expressed as means ± SEM. All statistical analyses were performed using R (R project). Comparisons of the two groups were done using a nonparametric bootstrap rank-sum test (Mann–Whitney *U* test). Comparisons of multiple groups were done by using ANOVA on ranks (Kruskal–Wallis *H* test) followed by a bootstrap *post hoc* test (pairwise Mann–Whitney *U* tests). For all statistical analysis, we used a bootstrap approach with 10,000 resampling and computed *p*-value, average difference and confidence interval. For clarity, we only include the *p*-value in the text. All parameters and results of the analysis are summarized in Table 1. A statistically significant difference was assumed with a *p*-value inferior to 0.05 and the mean difference confidence interval did not include 0.

**Table 1:** Summary of bootstrapped statistical analysis

Variable	*N* (core/shell)	Difference of mean	*p*-value	Confidence interval	
				Lower (2.5%)	Higher (97.5%)
Nuclei volume	39/33	−27.917	**0.022**	**−49.197**	**−5.654**
Nucleoli density	39/33	0.284	0.22	−0.004	0.622
Nucleoli volume	10/10	0.888	**0.01**	**0.282**	**1.513**
Stigmoid body volume	10	0.128	0.725	−0.7676	0.514
Stigmoid body diameter	10	0.092	0.547	−0.429	0.241
Somatic synapses	33/27	−1.990	0.029	−5.909	2.148
Proximal dendrites synapses	83/56	−5.290112	0.0488	−14.488749	4.118613
Soma mRGCs synapses	33/27	−2.79798	**9e-04**	**−4.632997**	**−1.003367**
Soma nonretinal synapses	33/27	1.124579	0.556	−1.437879	4.030471
Proxi dendrites mRGCs synapses	83/56	−9.194258	**1e-04**	**−14.714626**	**−3.669399**
Proxi dendrites nonretinal synapses	83/56	3.424585	0.6242	−2.507282	9.402726
DDCS linear density	5/11	−0.891	0.895	−2.762	0.325
DDCS vesicles	10/8	−217.927	0.160	−459.158	15.78672
Core mRGCs synapses (DDCS+/DDCS−)	25/11	8.674	**0.003**	**2.560**	**15.802**
Shell mRGCs synapses (DDCS+/DDCS−)	27/5	11.536	**0.005**	**5.305**	**18.467**
Core nonretinal synapses (DDCS+/DDCS−)	25/11	13.288	**0.003**	**6.251**	**20.385**
Shell nonretinal synapses (DDCS+/DDCS−)	27/5	14.506	**0.003**	**7.001**	**23.164**
Myelinated axon length	83/54	10.104	**0.006**	**2.397**	**17.905**
mRGCs axon length	50/50	1.469	0.721	−10.725	13.725
Nonretinal axon length	50/50	3.731	0.787	−6.710	14.325
mRGCs bouton linear density	50/50	−1.492	0.153	−4.194	1.243
Nonretina bouton linear density	50/50	1.172	0.360	−1.688	4.596
Bouton volume (mRGC/nonretinal without)	50/50	−0.5451	**6e-4**	**−0.938**	**−0.200**
Bouton volume (nonretinal without/with)	50/50	−0.572	**5e-4**	**−0.996**	**−0.122**
Mitochondria number (mRGC/nonretinal without)	50/50	−0.675	0.026	−1.475	0.075
Mitochondria number (nonretinal without/with)	50/50	−0.925	**0.003**	**−1.775**	**−0.025**
Synapse per bouton (mRGC/nonretinal without)	50/50	−0.2	0.185	−0.525	0.125
Synapse per bouton (nonretinal without/with)	50/50	−0.35	**0.031**	**−0.675**	**−0.025**
Vesicles density (mRGC/nonretinal without)	50/50	281.296	**0.018**	**107.597**	**468.311**
Vesicles density (nonretinal without/with)	50/50	430.199	**4.8e-10**	**314.409**	**543.212**
Mitochondria volume (mRGC/nonretinal without)	71/98	−0.009	0.2292	−0.023	0.004
Mitochondria volume (nonretinal without/with)	98/135	−0.015	**3.1e-5**	**−0.028**	**−0.002**

Bold values indicate significant difference.

## Results

### SBEM of mouse SCN subregions receiving mRGC inputs labeled with genetically encoded EM tags

**Figure 1.**
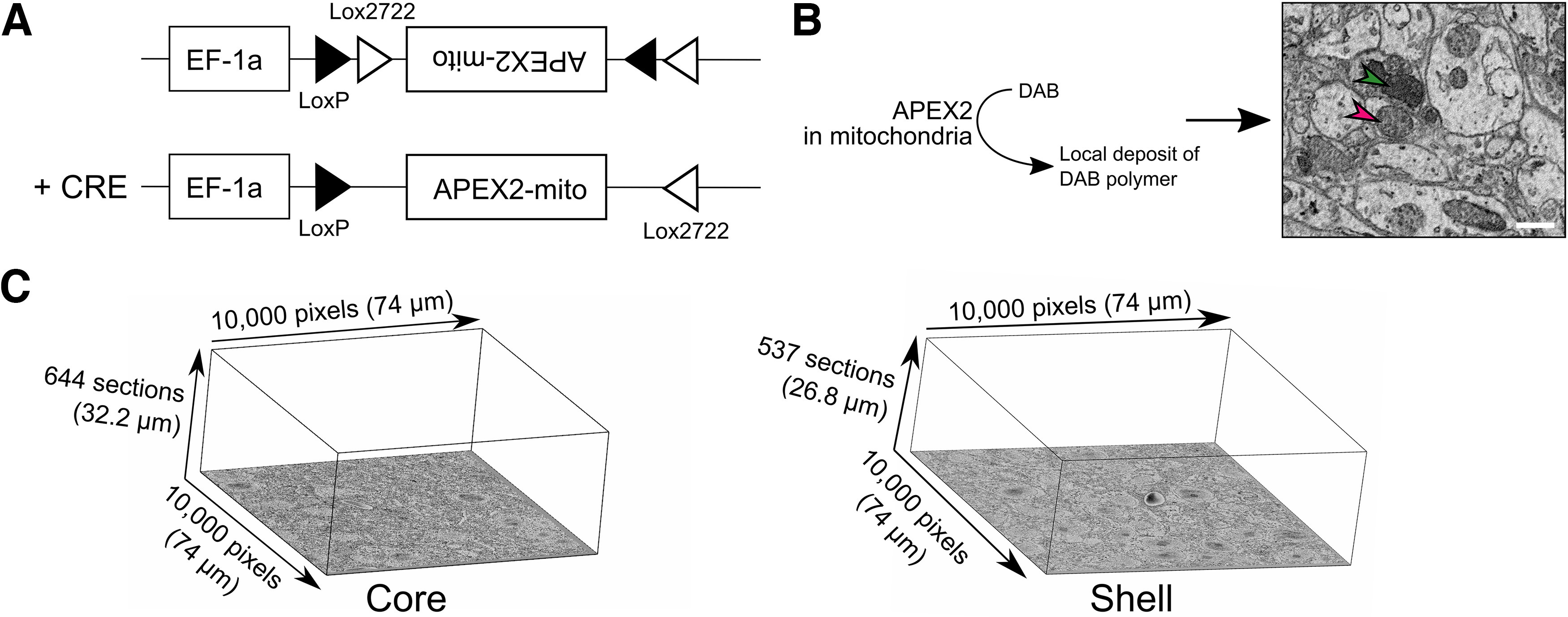
APEX2 specifically labels mRGCs mitochondria. ***A***, Construction of the Cre-dependent APEX2 vector. ***B***, Enzymatic oxidation of DAB by APEX2 forms a local deposit of DAB polymer in mitochondria that appear dense to electrons in EM images (red arrowhead = not labeled mitochondria, green arrowhead = labeled mitochondria; scale bar = 1 µm). ***C***, Representative image of the size of core and shell volumes.

To comprehensively study the ultrastructure of the mouse SCN, we used a method established in our lab to intravitreally inject an adeno-associated viral vector encoding an EM reporter, which is expressed in the presence of Cre-recombinase in melanopsin-expressing retinal ganglion cells (Opn4^Cre/+^ mice; (Hatori et al., 2008). Unlike our previous study (Kim et al., 2019), in which we used a plasma membrane delimited EM reporter, mini-SOG, followed by photo-oxidation of the tissue block, here we use a different EM reporter, APEX2 (AAV-EF1α-DIO-mito-V5-APEX2). The tag mito-V5 targets APEX2 to the mitochondrial membrane (Lam et al., 2014), thereby enabling the recognition of mRGCs axons in the SCN without labeling axon membrane. This facilitated the identification of synapses (Fig. 1; Extended Data Fig. 1-1). The use of this reporter does not require photo-oxidation, thus, is easier to stain and unlike membrane delimited mini-SOG, preserves the ultrastructural details of the labeled axons. These features allow large-scale staining (Lam et al., 2014; Martell et al., 2012) of neural tissues without toxicity.

10.1523/ENEURO.0227-23.2023.f1-1 Extended Data Figure 1-1 Examples of synapses in the SCN. Representative examples of EM images of DDCS (left) and boutons (right). Red arrowhead = DDCS; blue arrowhead = ADCS; orange arrowhead = dendritic intrusion; D = dendrite; B = bouton. Scale bar = 1 µm. Download Figure 1-1, TIFF file.

We co-injected our EM reporter with the Cre-dependent reporter DIO-tdTomato (AAV-EF1α-DIO-TdTomato), which is compatible with fluorescent microscopy, into both eyes of Opn4^Cre/+^ mice. Four weeks postinjection, we verified the comprehensive labeling of mRGCs in both eyes and then processed the animal’s brain for SBEM as described (Lam et al., 2014). Coronal brain sections of hypothalamic regions were scanned for the SCN mid-way along the rostro-caudal axis. The SCN was then divided into core and shell regions, as defined by the ventromedial SCN, and adjacent dorsolateral SCN, respectively. Each region was isolated and processed for SBEM. We collected 644 50-nm sections for the core and 537 sections for the shell. We choose a 50-nm section size to minimize the overlap of small structures (e.g., synaptic vesicles that usually have a diameter of ∼30 nm) in adjacent sections. We obtained a resolution of 7.4 nm per pixel.

In addition to the ultrastructural features of cells in the SCN, we found axonal processes that contained darkly stained mitochondria, which were most likely from mRGCs. As an initial random characterization of these axons with densely labeled mitochondria, we divided the volume on a five-by-five grid, resulting in 25 boxes. Nine boxes were randomly selected and 5–10 axons per box with darkly stained mitochondria were traced and segmented for their entire length within the volume. None of these axons traced back to a soma within the imaged volume. In addition, none were myelinated, as expected for mRGC axons, based on our previous work. All axons contained more than one labeled mitochondrion. However, among axons containing labeled mitochondria, nearly one-fourth (core = 22.92%; shell = 25.45%) had at least one unlabeled mitochondrion. In other words, based on mitochondria staining in one section alone, we cannot conclusively classify an axon as mRGC or nonretinal axon. Therefore, for all subsequent analyses, we traced all axons and checked for labeled mitochondria to classify them as an mRGC axon. Axons with only unlabeled mitochondria are likely of local origin (constituting the intra-SCN network) or afferent projections from nonretinal sources.

### Density and characteristics of SCN neurons in the core and the shell

**Figure 2.**
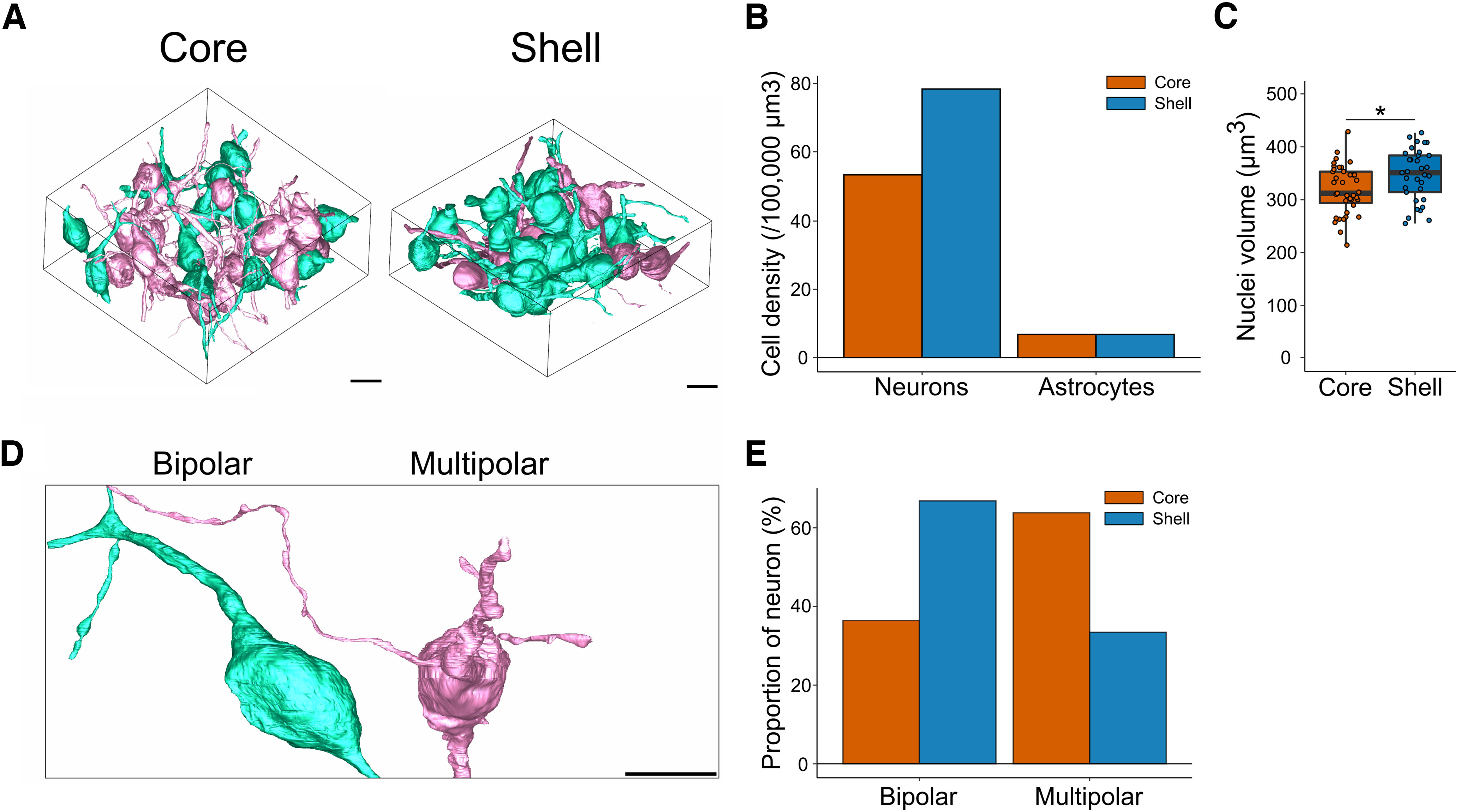
Density and types of SCN neurons. ***A***, Representative image of 3D reconstruction of all complete neurons in the core (left) and the shell (right). ***B***, Cell density of neurons and astrocytes. ***C***, Volume of SCN neurons nuclei in the core and shell. ***D***, Representative 3D reconstruction of unipolar, bipolar, and multipolar neurons (scale bar = 10 µm). ***E***, Proportion of unipolar, bipolar, and multipolar neurons in the core and the shell; *p < 0.05.

To evaluate the characteristics of cells in the core and shell of the SCN, we first segmented all cells whose nuclei were visible in the respective volumes (Fig. 2*A*). We found a slightly higher density of neurons in the shell compared with the core (core: 53.3 neurons per 100,000 µm³; shell = 67.5 per 100,000 µm³; Fig. 2*B*). We traced and reconstructed the nuclei and computed their volume. Nuclei were larger in the shell (core = 318.53 ± 7.35 µm^3^; shell = 346.45 ± 8.53 µm^3^; *p* < 0.05; Fig. 2*C*). We marked the astrocytes based on their characteristic features and found a similar density in both SCN regions (core: 6.7 glial cells per 100,000 µm^3^; shell: 5.7 glial cells per 100,000 µm^3^; Fig. 2*B*). Too few astrocyte nuclei were fully inside the volumes for a meaningful comparison of their volume or other characteristics.

SCN neurons have been characterized previously based on morphology parameters, especially on the number of neurites (van den Pol, 1980). We thus comprehensively segmented the processes of neurons whose soma was fully inside the volume (core = 26 neurons; shell = 20 neurons). Based on primary neurites extending from the soma, we classified neurons as unipolar, bipolar, or multipolar (Fig. 2*D*), as described previously (van den Pol, 1980). SCN neurons were either bipolar or multipolar with more bipolar neurons in the shell (core: 34.6%; shell: 60%) and more multipolar neurons in the core (core: 61.5%; shell: 35%, Fig. 2*E*). These differences in proportion could be explained by the presence of different populations of cells in each SCN region.

**Figure 3.**
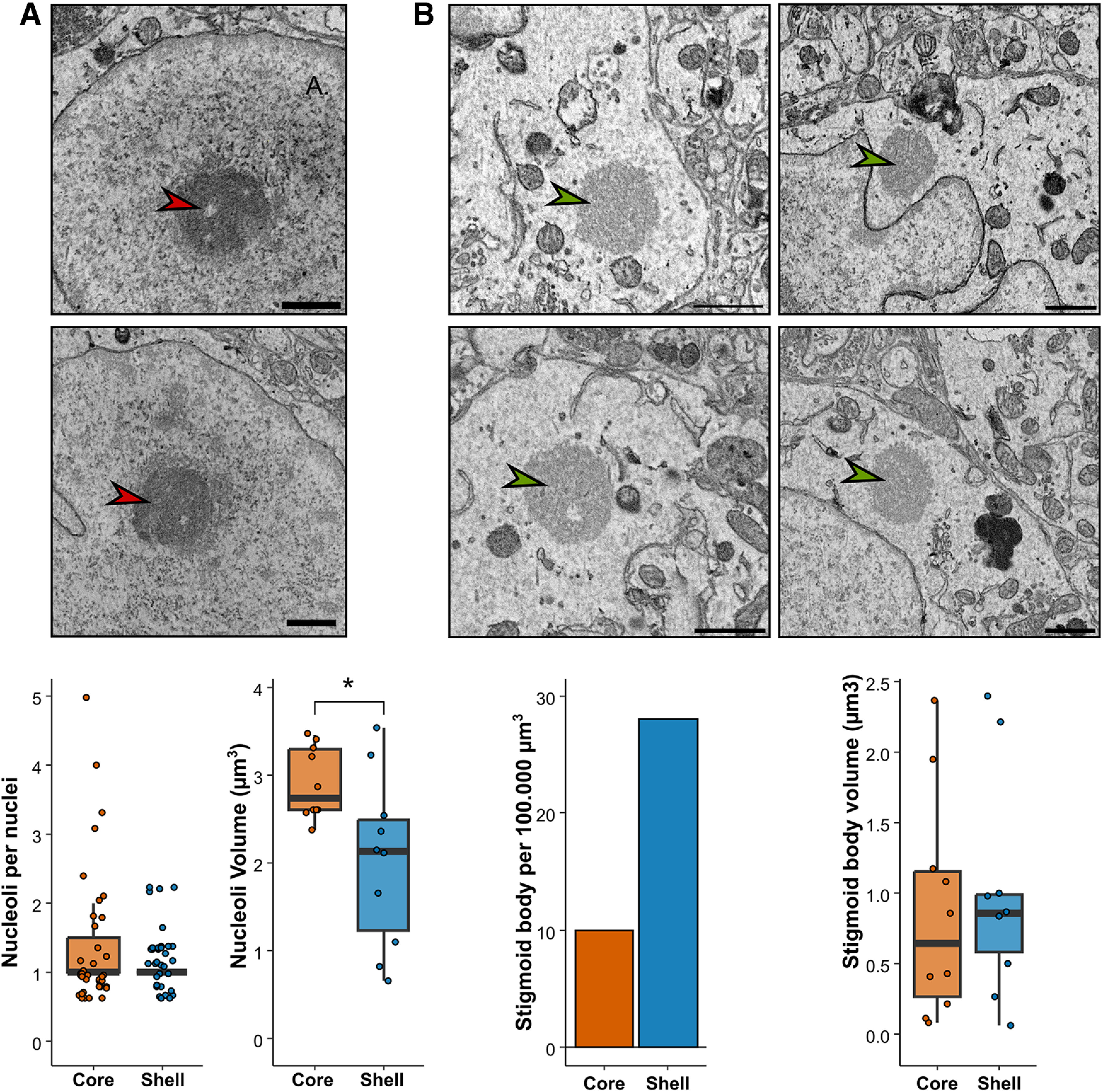
Nucleoli and stigmoid bodies in the core and the shell regions of the SCN. ***A***, Representative EM image (red arrows upper panel), linear density and volume of nucleoli per nuclei (lower panel) in the core and the shell. ***B***, Representative EM image (green arrows, upper panel), density and volume of stigmoid bodies (lower panel) in the core and the shell.

To further characterize neurons in both regions, we compared the density of nucleoli, a sign of high protein synthesis in the nucleus, and stigmoid bodies, a cytoplasmic structure specific to hypothalamic regions of the brain. We found a similar density of nucleoli in neurons in the core (1.44 ± 0.15 nucleoli/nuclei) and the shell (1.15 ± 0.06 nucleoli/nuclei, *p* = 0.219; Fig. 3*A*). However, nucleoli were bigger in the core (core = 2.91 ± 0.12 µm^3^; shell = 2.13 ± 0.30 µm^3^; *p* = 0.014). In the core, there were ∼10 stigmoid bodies per 100,000 µm³; in the shell, there were 28 stigmoid bodies per 100,000 µm³ (Fig. 3*B*). Despite this difference in density, stigmoid bodies had a similar volume (core = 0.86 ± 0.24 µm^3^; shell = 0.99 ± 0.24 µm^3^; *p* = 0.725).

### SCN neurons in the core and shell receive dense somatic and proximal synaptic input

**Figure 4.**
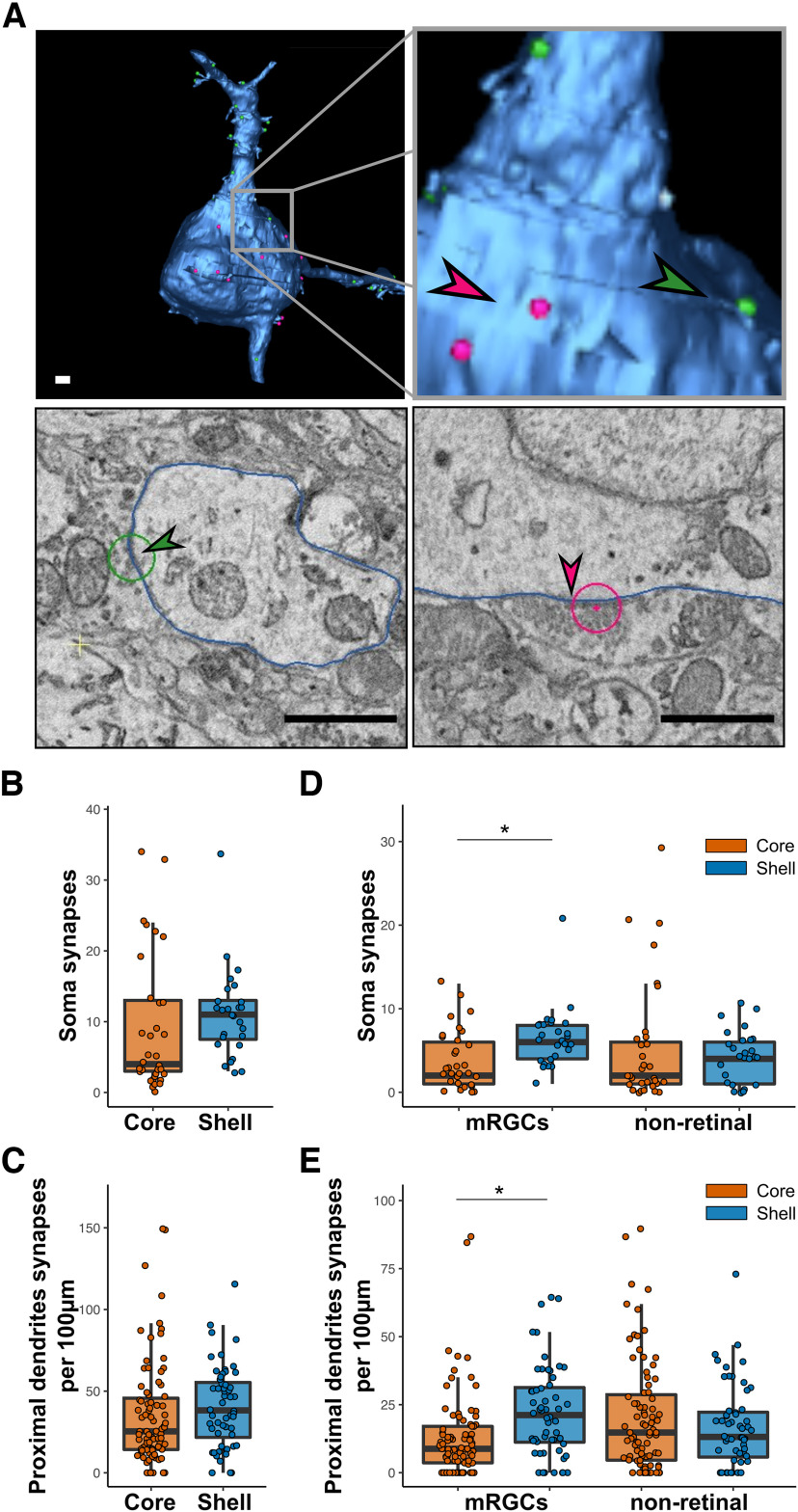
Proximal connectivity of SCN neurons. ***A***, Representative image of 3D reconstruction of a neuron (upper panel) and EM images of synapses (lower panel) on proximal dendrite (green arrow, left) and on soma (red arrow, right). Scale bar = 1 µm. ***B***, Total number of synapses contacting the soma. *N* = 27–33. ***C***, Total number of synapses contacting proximal dendrites. *N* = 56–83. ***D***, Number of mRGCs and nonretinal synapses contacting the soma. *N* = 27–33. ***E***, Number of mRGCs and nonretinal synapses contacting proximal dendrites. *N* = 56–83; *p < 0.05.

As the core and shell of the SCN contained different subtypes of neurons, we evaluated the number of synapses on the soma and proximal axons of SCN neurons to see whether one type of neuron receives more mRGC synaptic input. Somas and proximal dendrites were fully segmented (core = 33 neurons; shell = 27 neurons). Then we systematically inspected the cell membranes of these neurons in all sections of the volumes to identify synapses. Neurites with a constant small diameter were classified as axons and excluded from the analyses. None of these axons made or received synapses in either volume. We found 891 synapses in the core and 764 in the shell that contacted the soma or proximal dendrites (Fig. 4*A*). We found no difference in the density of synapses on the soma (core = 9.12 ± 1.68 synapses; shell = 11.11 ± 1.21; Fig. 4*B*) or on proximal dendrites (core = 37.27 ± 3.80 synapses/100 µm; shell = 40.62 ± 4.01 synapses/100 µm; Fig. 4*C*).

We then refined the analysis by using the APEX2 labeling to classify the synapses as being associated with mRGCs (with APEX2 labeled mitochondria) or nonretina axons. We found that the number of mRGC synapses on the soma was higher in the shell than in the core (core = 3.76 ± 0.64 synapses; shell = 6.56 ± 0.70; *p* < 0.001; Fig. 4*D*), as well as the linear density of synapses on proximal dendrites (core = 12.32 ± 1.68 synapses/100 µm; shell = 22.08 ± 2.61 synapses/100 µm; *p* < 0.001; Fig. 4*E*). By contrast, we found no difference in the nonretinal synaptic density on the soma (core = 5.27 ± 1.27 synapses; shell = 4.15 ± 0.61; *p* = 0.55; Fig. 4*D*) or proximal dendrites (core = 18.71 ± 2.71 synapses/100 µm; shell = 16.91 ± 2.15 synapses/100 µm; *p* = 0.62; Fig. 4*E*).

Finally, we used the classification of neurons established earlier as bipolar or multipolar to verify whether a particular subtype of neurons received more of one type of connection. The synaptic density was homogeneous among all groups in both volumes (Extended Data Fig. 4-1).

10.1523/ENEURO.0227-23.2023.f4-1 Extended Data Figure 4-1 Synaptic densities on soma, proximal dendrites and distal dendrites. ***A***, ***B***, Total synapse density on soma (***A***) and proximal dendrites (***B***) of bipolar and multipolar neurons in the core and the Shell. ***C***, ***D***, Density of mRGCs and nonretinal synapses on the soma (***C***) and proximal dendrites (***D***) of bipolar and multipolar neurons in the core and the shell. ***E***, Linear density of ADCS on DDCS-positive and negative dendrites. ***F***, Average length of DDCS-positive and negative dendrites; *p < 0.05. Download Figure 4-1, TIFF file.

### SCN neurons form a dense dendro-dendritic network of chemical synapses

In addition to the direct synaptic contacts on somas and proximal dendrites, we further studied the density of axodendritic chemical synapses (ADCSs) on distal dendrites, defined as dendrites from a neuron whose soma was not within our collected volume, and the presence of dendro-dendritic chemical synapses (DDCSs). We previously showed that DDCS form a network between SCN neurons (Kim et al., 2019).

**Figure 5.**
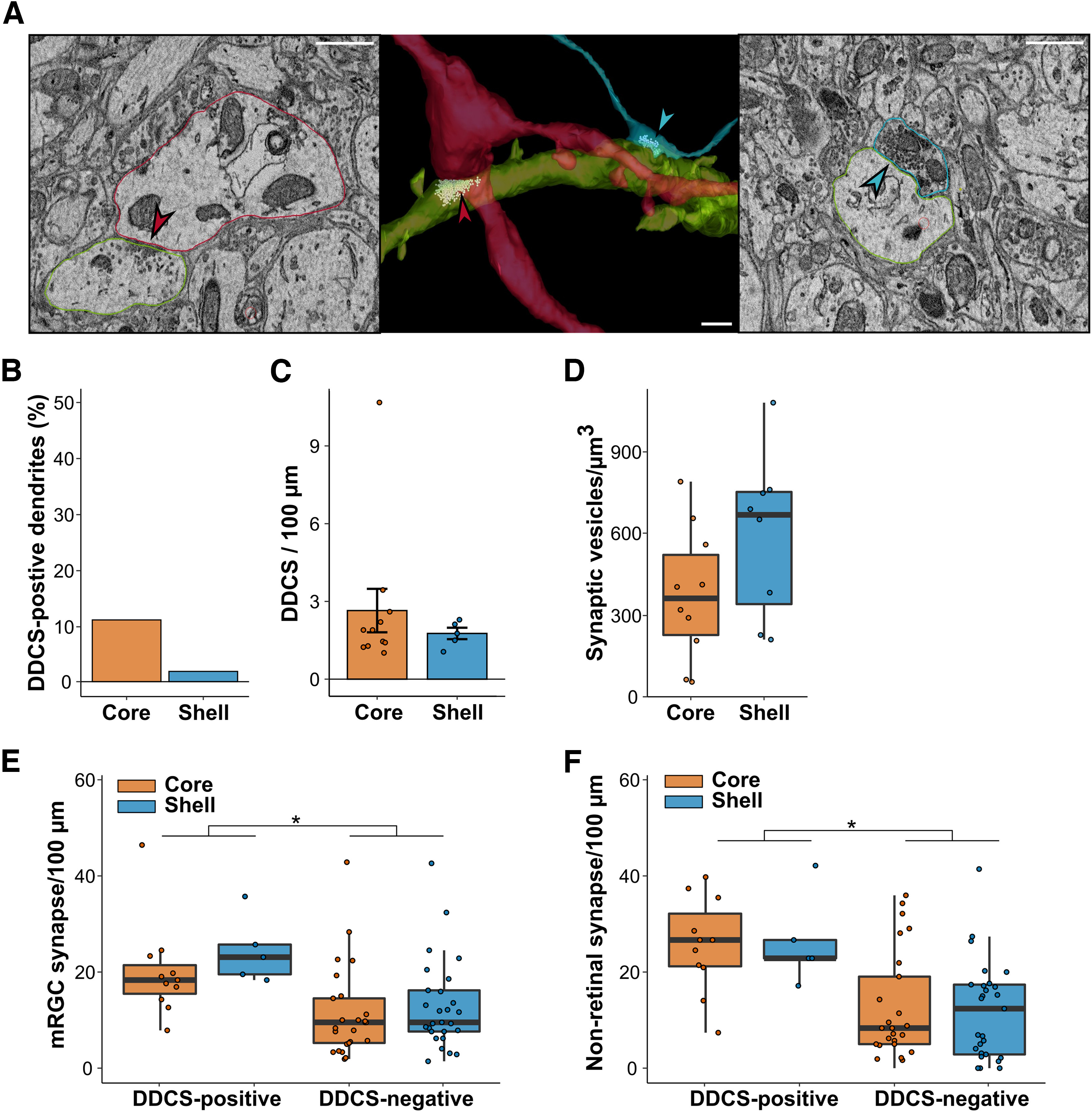
Network of dendro-dendritic synapses in the SCN. ***A***, 3D reconstruction (middle) and representative image of EM image of DDCS (left, red arrow) and ADCS (right, blue arrow). Scale bar = 1 µm. ***B***, Percentage of dendrites forming at least one DDCS. ***C***, Linear density of DDCS along DDCS-positive dendrites. *N* = 5–11. ***D***, Synaptic vesicle density at the proximity of DDCS. *N* = 5–11. ***E***, Linear density of mRGCs synapses on DDCS-positive and negative dendrites. *N* = 5–27. ***F***, Linear density of nonretinal synapses on DDCS-positive and negative dendrites. *N* = 5–27; *p < 0.05.

To estimate the density of the DDCS network in the two regions of the SCN, we randomly selected 100 dendrites from the core and shell volumes. We fully skeletonized them and searched for synapses where the associated element (presynaptic or postsynaptic) was also a dendrite (see Methods for the criteria of identification used; Fig. 5*A*; Extended Data Fig. 1-1). In the core, 10.6% of dendrites have at least one DDCS, whereas only 1.9% of dendrites in the shell form DDCS (Fig. 5*B*). However, we found a similar linear density of DDCS in DDCS-positive dendrites in the core and shell (core = 2.64 ± 0.83 DDCS/100 µm, shell = 1.75 ± 0.22 DDCS/100 µm; *p* = 0.79, Fig. 5*C*). DDCS were identified previously in the SCN, but we took advantage of SBEM to characterize them further. Dendrites with DDCS did not show any morphologic changes close to the DDCS. The contact between the two dendrites was usually directly on the dendrite shaft. One occurrence of a DDCS on a dendritic spine was observed in our volume. The presynaptic element of DDCS were surrounded by smooth endoplasmic reticulum and at least one mitochondrion within 1 µm. The density of synaptic vesicles around the DDCS was not higher in the shell compared with the core (core = 375.34 ± 76,78 synaptic vesicles/µm^3^, shell = 593.26 ± 106.16 vesicles/µm^3^; *p* = 0.15; Fig. 5*D*).

Among DDCS-positive dendrites, we determined the linear density of ADCS with mRGCs and nonretinal axons. The same analyses were conducted on a similar number of dendrites that do not form DDCS (DDCS-negative dendrites). We counted 720 synapses in the core and 501 in the shell. DDCS-positive dendrites received a higher number of synapses than DDCS-negative dendrites in both the core and shell (DDCS-positive: core = 53.24 ± 5.45 synapses/100 µm, shell = 57.08 ± 7.14 synapses/100 µm; DDCS-negative: core = 25.52 ± 3.23 synapses/100 µm, shell = 25.50 ± 2.89 synapses/100 µm, *p* < 0.001; Extended Data Fig. 4-1), despite having a similar total length (DDCS-positive: core = 67.45 ± 6.25 µm, shell = 70.03 ± 8.84 µm, *p* = 0.8269; DDCS-negative: core = 54.28 ± 2.94 µm, shell = 47.20 ± 2.95 µm, *p* = 0.099; Extended Data Fig. 4-1). After classifying synaptic boutons as mRGCs or nonretinal, we observed a similar pattern. The synaptic density was similar in the core and shell, but higher for DDCS-positive dendrites compared with DDCS-negative dendrites for both mRGCs ADCS (DDCS-positive: core = 20.10 ± 2.97, shell = 24.46 ± 3.11; DDCS-negative: core = 11.43 ± 1.90, shell = 12.93 ± 1.79, *p* < 0.001; Fig. 5*E*) and nonretinal ADCS (DDCS-positive: core = 25.75 ± 2.93, shell = 26.32 ± 4.21; DDCS-negative: core = 12.46 ± 2.24, shell = 11.82 ± 1.96, *p* < 0.001; Fig. 5*F*). A small fraction of boutons forming synapses with these dendrites (∼5%) were not identified as either mRGCs or nonretinal because they did not have visible mitochondria. These boutons were mostly close to the edge of the volume, reducing our capacity to follow the axon to find mitochondria and identify them.

In summary, dendrites forming DDCS receive similar synaptic input from mRGCs and nonretinal axons in the core and the shell and receive more synaptic input than DDCS-negative dendrites. DDCSs are, however, more frequent in the core, suggesting a denser network of interconnected dendrites/neurons compared with the shell.

### Myelinated and nonmyelinated axons in the SCN

**Figure 6.**
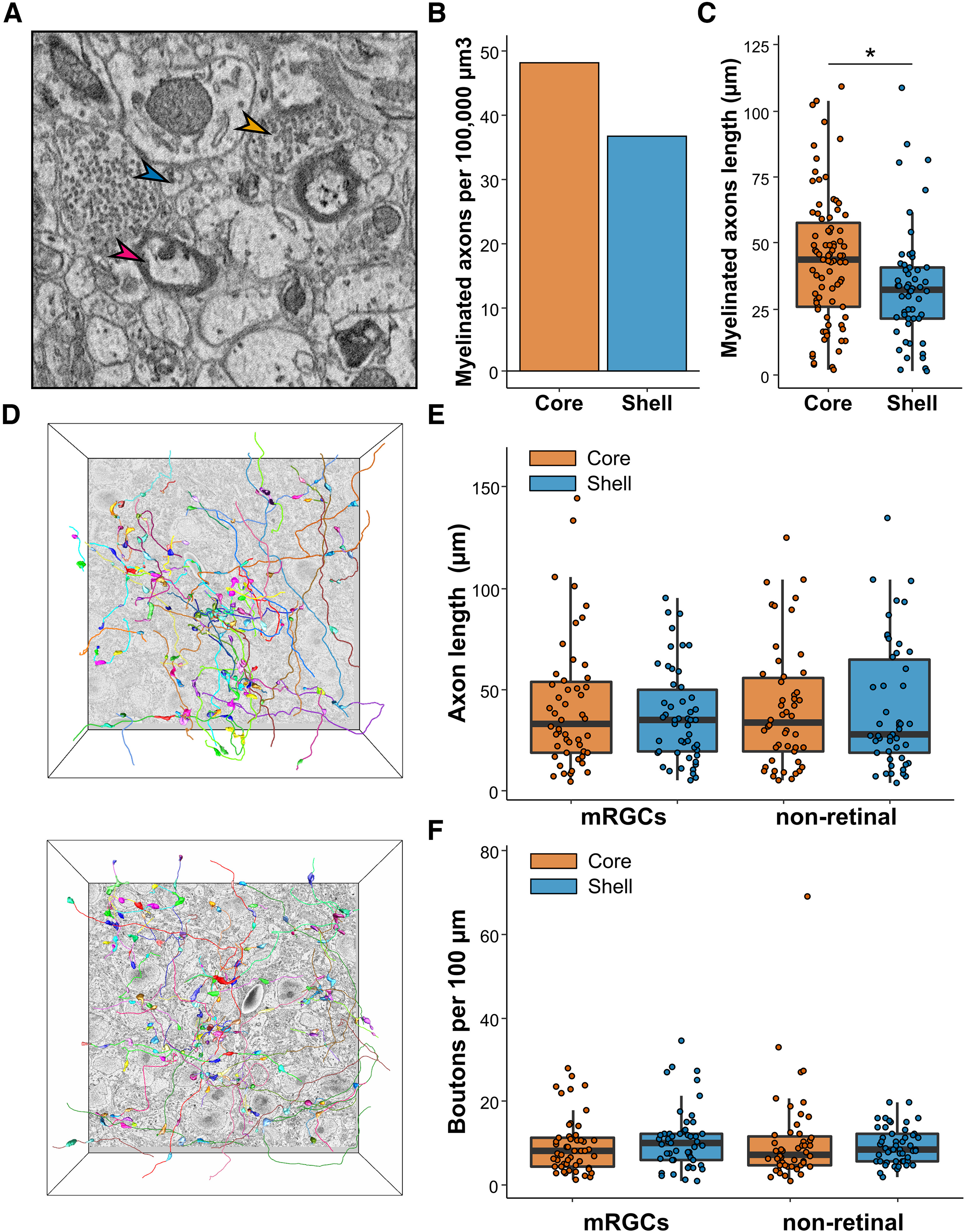
Myelinated and nonmyelinated axons. ***A***, Representative image of axons found in the SCN. Myelinated axons (red arrow), nonmyelinated axons (blue arrow), and axonic boutons (orange arrow). ***B***, Density of myelinated axons in the SCN volumes. ***C***, Length of myelinated axons in the SCN. *N* = 83–54. ***D***, Representative image of the fully segmented axons and boutons in the core (upper panel) and the shell (lower panel). *N* = 50. ***E***, mRGCs and nonretinal axons length. *N* = 50. ***F***, Boutons linear density along mRGCs and nonretinal axons. *N* = 50; *p < 0.05.

Two types of axons are present in the SCN: myelinated and nonmyelinated (Fig. 6*A*). We fully segmented myelinated axons in both volumes and examined their density, length, and branching pattern. None were associated with APEX-labeled mitochondria and thus none originated from the retina. We identified 85 myelinated axons in the core and 54 in the shell. This corresponds to a slightly higher density of myelinated axons in the core compared with the shell (core = 48.21 axons/100,000 µm³, shell = 33.62 axons/100,000 µm³; Fig. 6*B*). There were longer segments of myelinated axons in the core compared with the shell (core = 43.10 ± 2.69 µm, shell = 33.62 ± 2.97 µm; *p* <0.01; Fig. 6*C*), possibly because of the proximity of the optic chiasma or their branching in the volume. In the shell, none of the 54 myelinated axons had a branch, whereas, in the core, four of the 85 myelinated axons branched once. In both volumes, only two axons with demyelinated parts were observed but they did not form any boutons or synapses. Myelinated axons did not appear to participate in the SCN connectome in our two volumes. They may connect to an adjacent brain region or a different part of the SCN.

Nonmyelinated axons, however, contributed greatly to the SCN connectome. To analyze the characteristics of nonmyelinated axons, we randomly selected 50 mRGCs and 50 nonretinal axons in each SCN volume, manually traced them, and quantified their length and bouton linear density (Fig. 6*D*). None of these axons traced back to a soma within our volume. We did not observe any difference in the average length of mRGCs versus nonretinal axons (mRGCs: core = 42.26 ± 4.52 µm; shell = 40.79 ± 4.40 µm, *p* = 0.7289; nonretinal: core = 43.51 ± 3.14 µm; shell = 36.66 ± 2.91 µm, *p* = 0.108; Fig. 6*E*) or the linear density of boutons along these axons (mRGCs: core = 9.45 ± 0.95 boutons/100 μm; shell = 10.93 ± 1.03 boutons/100 μm; *p* = 0.1577; nonretinal: core = 11.45 ± 1.31 boutons/100 μm; shell = 13.02 ± 1.42 boutons/100 μm; *p* = 0.791; Fig. 6*F*). The larger number of mRGCs contacts with DDCS-positive dendrites thus cannot be explained by a higher density of boutons or linear density of synapses. Instead, these data suggests that mRGCs exhibit a synaptic preference for DDCS-positive dendrites in both the core and shell of the SCN.

### mRGCs and nonretinal boutons have different morphologic characteristics

#### Density and volume of mRGCs and nonretinal boutons

**Figure 7.**
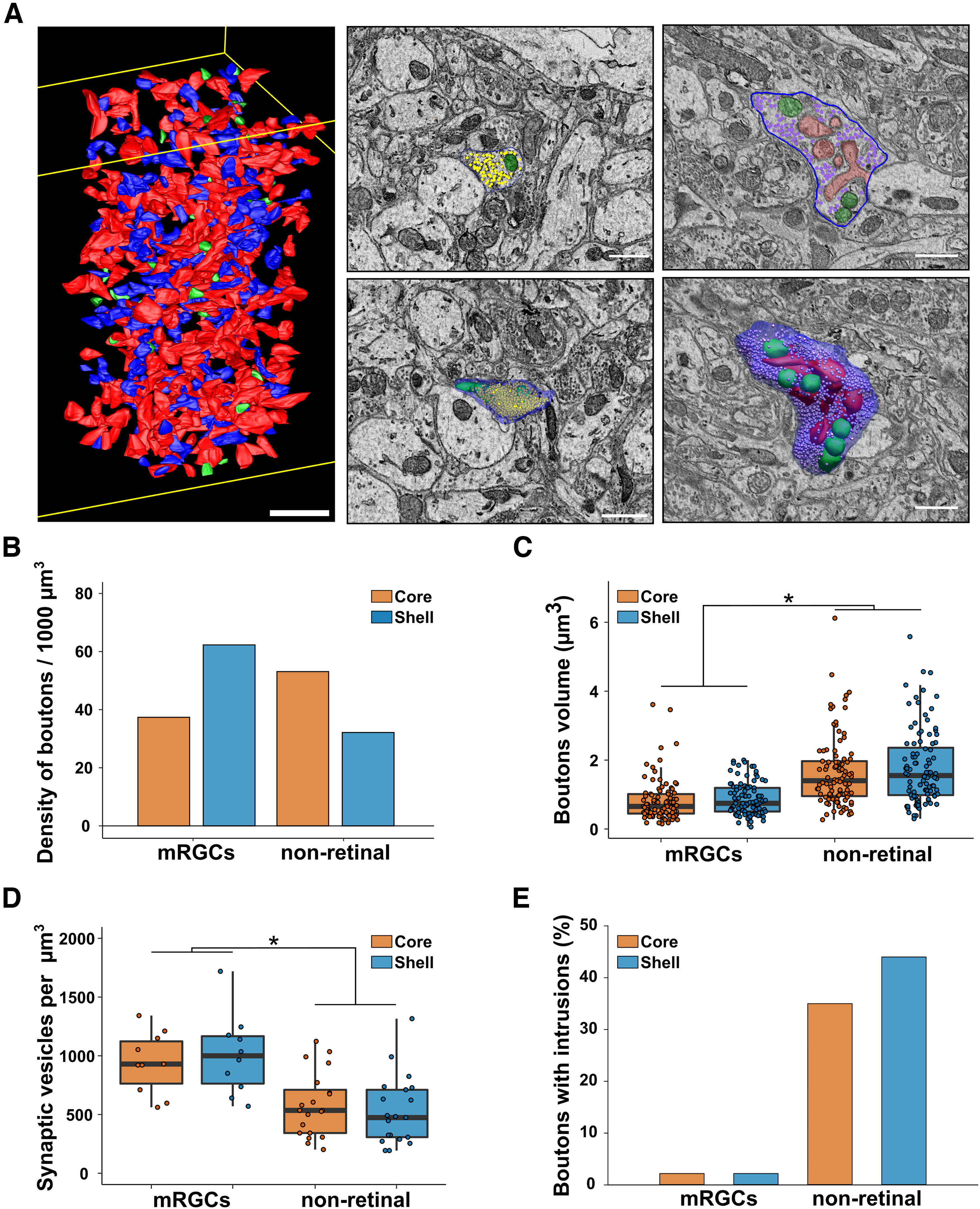
Characteristics of axonal boutons. ***A***, Example of a box with all boutons segmented (left, red = nonretinal boutons; blue = mRGCs boutons; green = nonidentified boutons; scale bar = 5 µm) and representative EM image and 3D reconstruction of boutons without (middle) or with (right) intrusions (green = mitochondria, red = dendritic intrusions, blue = cell membrane, dots = synaptic vesicles; scale bar = 1 µm). ***B***, Density of mRGCs and nonretinal boutons. ***C***, Volume of mRGCs and nonretinal boutons. *N* = 50. ***D***, Density of synaptic vesicles in mRGCs and nonretinal boutons. *N* = 10. ***E***, Percentage of mRGCs and nonretinal boutons containing intrusions. *N* = 50 *p < 0.05.

As we saw that synaptic input is not homogeneous in the SCN, we further characterized axonic boutons in the core and shell. We selected 1 box out of the five-by-five grid previously created for both SCN volumes. In this box we exhaustively searched for all boutons and labeled them as mRGCs or nonretinal boutons, depending on the presence of labeled mitochondria (Fig. 7*A*; Extended Data Fig. 1-1). A small fraction of boutons was not identified as they did not have mitochondria and were not connected to another bouton in the volume.

**Figure 8.**
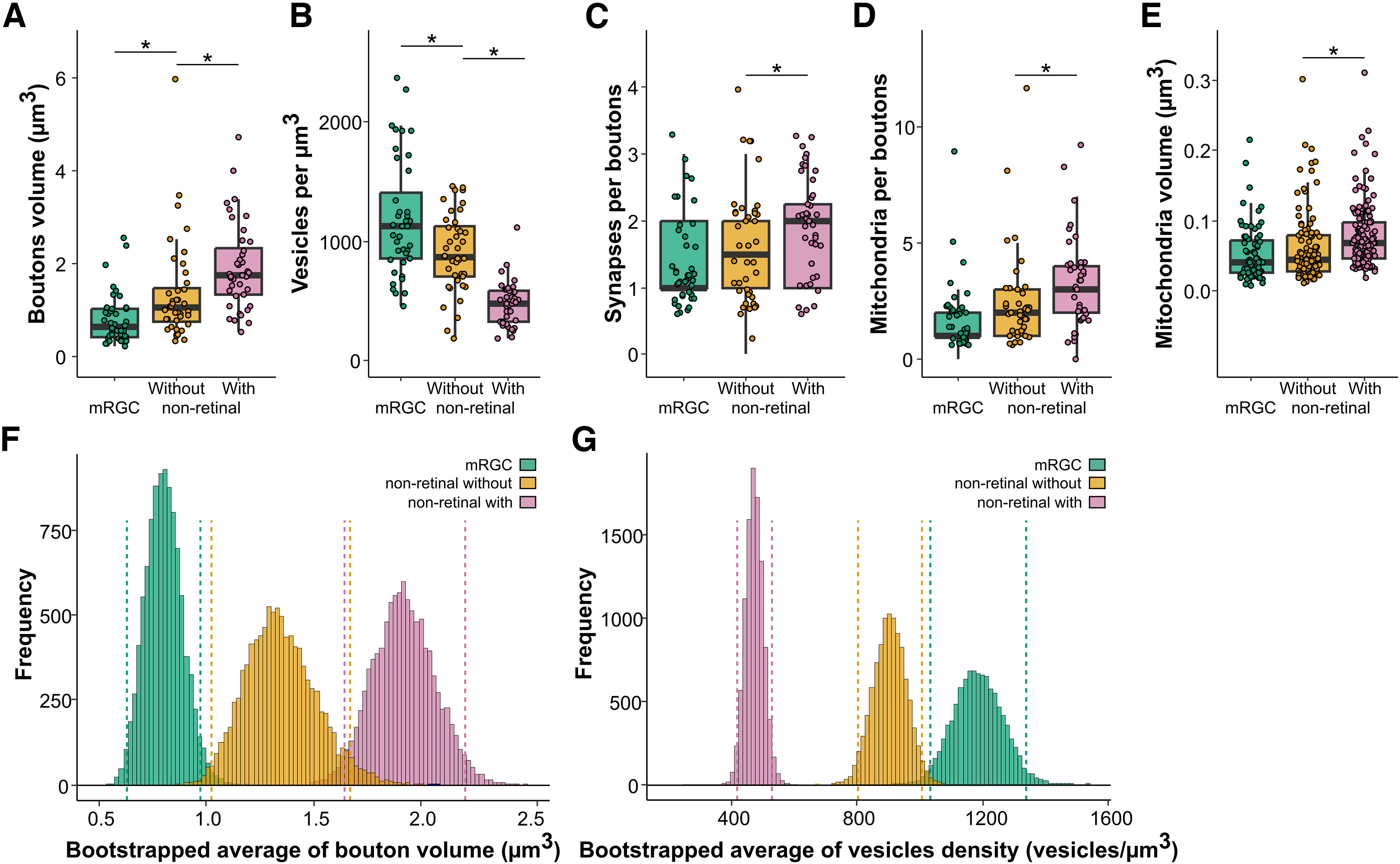
Characteristics of mRGCs and nonretinal boutons, with and without dendritic intrusions. ***A***, Volume of boutons (µm^3^). *N* = 50. ***B***,Density of synaptic vesicles in boutons. *N* = 50. ***C***, Number of synapses per bouton. *N* = 50. ***D***, Number of mitochondria per boutons. *N* = 50. ***E***, Volume of individual mitochondria in boutons. ***F***,***G***, Distribution of the bootstrapped average of bouton volume (***F***) and vesicle density (***G***) in mRGCs and non-retinal boutons. Dashed lines correspond to 2.5% and 97.5% of the respective distribution. *N* = 71–135; *p < 0.05.

In the core, we counted 675 boutons: 39.0% mRGC, 55.5% nonretinal, and 5.7% unidentified. In the shell, we counted 601 boutons: 61.1% mRGC, 31.4% nonretinal, and 7.4% nonidentified. Based on the volume of the boxes (core SCN = 7052 µm³; shell SCN = 5880 µm³), we obtained a similar density of boutons in the two regions (core = 95.71 boutons/1000 µm³; shell = 102.19 boutons/1000 µm³). However, the density of mRGCs boutons was greater in the shell than in the core (core = 37.29 boutons/1000 µm³, shell = 62.40 boutons/1000 µm³; Fig. 7*B*). By contrast, the density of nonretinal boutons was greater in the core than in the shell (core = 37.29 boutons/1000 µm³, shell = 62.40 boutons/1000 µm³; Fig. 7*B*).

#### Characteristics of mRGC and nonretinal boutons

We then randomly selected 50 mRGC and 50 nonretinal boutons and fully segment them and their content (Fig. 7*A*). We did not observe a difference in the volume of mRGC boutons (core = 0.83 ± 0.06 µm^3^; shell 0.88 ± 0.05 µm^3^; *p* = 0.12; Fig. 7*C*) or nonretinal boutons (core = 1.66 ± 0.10 µm^3^; shell 1.80 ± 0.11 µm^3^; *p* = 0.40; Fig. 7*C*) between the core and shell. However, nonretinal boutons were larger than mRGCs boutons in both regions (*p* < 0.001). By contrast, mRGCs boutons have a higher density of synaptic vesicles (core = 938.76 ± 82.14 synaptic vesicles/µm^3^; shell 1006.16 ± 107.34 synaptic vesicles/µm^3^; Fig. 7*D*) compared with nonretinal boutons (core = 580.66 ± 61.15 synaptic vesicles/µm^3^; shell 528.42 ± 65.69 synaptic vesicles/µm^3^; *p* < 0.001; Fig. 7*D*). We then noticed that nonretinal boutons were not morphologically homogeneous. Indeed, more than a third of nonretinal boutons had dendrites forming intrusions into them (core = 35%; shell = 44%; Fig. 7*E*). This morphology was almost nonexistent for mRGC boutons (<2%). These structures increase the contact surface between presynaptic and postsynaptic elements and present the characteristics of synapses.

Axonal boutons in the SCN can thus be categorized as either mRGC, nonretinal without intrusions, or nonretinal with intrusions. They could also be distinguished via their size, with mRGCs boutons being the smallest (mRGCs = 0.803 ± 0.087 µm^3^; nonretinal without = 1.348 ± 0.169 µm^3^; nonretinal with = 1.921 ± 0.147; *p* < 0.05; Fig. 8*A*). By contrast, mRGC boutons were more densely packed with vesicles (mRGCs = 1186.152 ± 77.195 vesicles/µm^3^; nonretinal without = 904.856 ± 52.225 vesicles/µm^3^; nonretinal with = 474.657 ± 28.733 vesicles/µm^3^; *p* < 0.05; Fig. 8*B*). However, mRGCs boutons and nonretinal boutons without intrusions did not differ based on the number of synapses (mRGCs = 1.425 ± 0.106 synapses/bouton; nonretinal without = 1.625 ± 0.127 synapses/bouton; *p* < 0.05; Fig. 8*C*), the number of mitochondria (mRGCs = 0.803 ± 0.087 mitochondria/bouton; nonretinal without = 1.348 ± 0.169 mitochondria/bouton; *p* < 0.05; Fig. 8*D*), or the volume of individual mitochondria (mRGCs = 0.052 ± 0.004 µm^3^; nonretinal without = 0.062 ± 0.005 µm^3^; *p* < 0.05; Fig. 8*E*). Nonretinal boutons with intrusions had more synapses (nonretinal with = 1.975 ± 0.115 synapses/bouton; *p* = 0.031; Fig. 8*C*), more mitochondria (nonretinal with = 1.921 ± 0.147 mitochondria/bouton; *p* = 0.003; Fig. 8*D*), and slightly larger mitochondria (nonretinal with = 0.078 ± 0.003 µm^3^; *p* < 0.001; Fig. 8*E*) compared with nonretinal boutons without intrusions.

The observed morphologic differences between boutons could mean they are associated with different types of axons (e.g., axons of different origins, SCN vs external). To test this hypothesis, we systematically assessed the types of boutons along the axons we previously skeletonized. As expected, mRGCs axons all had the same type of bouton in the core and shell. For nonretinal axons, the majority of axons had only one type of bouton. In the core, out of the 50 axons with multiple boutons checked, only three (6%) had a bouton different from the others. In the shell, five out of 50 axons (10%) had mixed bouton types.

These morphologic differences between mRGC and nonretinal boutons provide the possibility of establishing criteria for identifying boutons without specific labeling. Based on our results, in addition to the presence of dendritic intrusions, bouton volume, and synaptic vesicle density are the main parameters that distinguish between the different boutons types. We used a bootstrap resampling method to estimate the population average confidence interval for boutons volume and vesicle density. The confidence intervals of mRGC boutons did not overlap with nonretinal boutons for both bouton volume (mRGC = [0.6379;0.9758]; nonretinal without = [1.0270;1.677]; nonretinal with = [1.633;2.198]) and vesicle density (mRGC = [1036.0;1338.0]; nonretinal without = [802.8;1007.1]; nonretinal with = [419.0;529.6]). This means that for boutons with a volume between 0.64 and 0.98 µm^3^, there is a 95% chance that they are part of an mRGC axon.

## Discussion

We presented here the first analysis of the internal and external connectivity of the SCN at this resolution. SBEM allowed us to access at an unprecedented scale how retinal input is distributed between the two major subregions of the SCN, revealing both similarities and disparities.

### SCN neurons

Our results show that the shell region of the SCN is more densely packed with neurons than the core region. Previously, it was shown the shell has a larger volume with a lower cell density compared with the core which was later confirmed later with cell counting (van den Pol, 1980; Moore et al., 2002). Our results support this observation. The density of astrocytes estimated from the current volumes (∼70,000 astrocytes per mm^3^) is close to the number obtained from the SCNs of male rats (68,627 astrocytes per mm^3^; Güldner, 1983). The ratio of neurons to glial cells of ∼10:1 is higher than in other brain regions. Glial cells have a critical role in the rhythmic function of the SCN (Brancaccio et al., 2019). Unfortunately, the current volumes contained too few glial cells to conduct a meaningful study of this cell type in the SCN.

We classified SCN neurons into three subtypes based on the number of neurites (unipolar, bipolar, and multipolar), which is a simplified version of the classification introduced by van den Pol in 1980. Our results partially match their findings as we did not observe unipolar cells, only bipolar or multipolar cells, and the latter was more frequent in the core versus the shell. It has been suggested that these morphologically different neurons support specific electrophysiological functions (Jiang et al., 1997). That study found that bipolar cells are more likely innervated by the retinohypothalamic tract and to be efferent cells. Radial multipolar neurons are the only ones that did not respond with EPSCs to optic nerve stimulation and are thought to be SCN interneurons (Jiang et al., 1997). Again, our findings only partially confirm these hypotheses. We observed mRGC synaptic inputs on both types of neuronal cells (bipolar and multipolar), either on the proximal dendrite or soma. These morphologic subtypes of SCN neurons could correspond to the different populations of neurons that express different neuropeptides. Initially, it was thought that neurons receiving the majority of retinal input were VIP-expressing neurons (Lokshin et al., 2015). A subsequent study evaluated the retinal input to the soma of the cells expressing the three main neuropeptides (vasoactive intestinal peptide, arginine vasopressin, and gastrin releasing peptide) and conclude that all cell types receive denser synaptic input from nonretinal cells (Fernandez et al., 2016). Our data show that retinal input accounts for up to 40% of synaptic contacts on SCN neurons. Previous studies were performed using light microscopy, which has a lower resolution than in the present study, and could have led to an underestimation of synaptic input on the soma by including synapses on proximal dendrites.

Most interestingly, we observed a higher density of mRGC synapses in the shell compared with the core. Fernandez and colleagues (Fernandez et al., 2016) showed that neurons in the shell require binocular light stimulation to induce C-Fos, which could explain why the synaptic density is almost double in the shell.

### Anatomy of boutons depends on axon origin

The SCN core mainly receives axonal projections from the intergeniculate leaflet (IGL) and the pretectal nuclei, whereas the SCN shell receives inputs from limbic, hypothalamic, and brainstem nuclei (Abrahamson and Moore, 2001). In addition, both regions of the SCN receive photic information from the retina through mRGCs (Hattar et al., 2006; Göz et al., 2008; Güler et al., 2008; Hatori et al., 2008). The present study reveals for the first time the detailed architecture of mRGC axons in the two subregions of the SCN. In addition to their different bouton volumes, nonretinal and mRGC boutons show several morphologic specificities. Although both types of boutons contain a generic profile of synaptic organelles visible in EM images (mitochondria, vesicles, and endoplasmic reticulum), the main difference is the presence of dendritic intrusions. Close to half of nonretinal boutons in multiple sets of samples contained intrusions of dendritic spines, whereas mRGC boutons in the SCN contained almost none. At the point of contact between these synaptic boutons and intrusions, some small synaptic vesicles were present.

According to previous literature on the origins of axons innervating the SCN, big vesicles could contain serotonin (5-HT) from raphe nuclei afferents (Pelletier et al., 1981; Pickel and Chan, 1999) or neuropeptide Y (NPY) from the geniculohypothalamic tract (GHT; Card and Moore, 1982; Harrington et al., 1987; van den Pol, 2012). Small vesicles could contain γ-aminobutyric acid (GABA) from other SCN neurons or the GHT (Deken et al., 2003; Silver and Rainbow, 2015). To identify a synapse as excitatory or inhibitory, the gold standard in EM is to use specific features: excitatory synapses should have a dark postsynaptic density (“asymmetric synapse”), have small round vesicles, and preferentially target dendritic spines, whereas inhibitory synapses should be symmetric (no postsynaptic density), have elongated synaptic vesicles, and rarely target dendritic spines (Uchizono, 1965; Colonnier, 1968; Peters and Webster, 1991). We were not able to use these criteria in our dataset, as we were not able to identify some elements. For example, we were not able to reproducibly identify postsynaptic densities, even for mRGC synapses that are known to be primarily glutamatergic with a small population of GABAergic synapses (Castel et al., 1993; Purrier et al., 2014; Sonoda et al., 2020). We, therefore, decided not to use this classification in this study. Nonetheless, even without this characterization, our data still provide useful insights into the connectivity of the SCN.

The comparison of mRGC bouton between core and shell SCN reveals the different synaptic strengths between mRGCs in different parts of SCN. Synaptic strength can be assessed both by current strength and subsynaptic structures, such as the number of mitochondria near the synaptic cleft (Verstreken et al., 2005), the number and distribution of synaptic vesicles (Karunanithi et al., 2002), bouton size/volume, and more (Meyer et al., 2014). Our data show that the shell has a slightly higher density of boutons than the core, while the average number of mRGC boutons on each axon is not different between the core and shell SCN (given that axon lengths and branching patterns are similar). Together, these data reveal equally dense mRGC projections into both regions of the SCN and a stronger synaptic connection between mRGCs and the SCN shell. This should be evaluated further. In agreement with our observation, previous studies have shown direct retinal innervation of both the core and shell SCN (Hattar et al., 2006; Beier et al., 2020). By contrast, it has been reported that the core SCN receives most of the retinal input and transfers this input to the shell SCN (Abrahamson and Moore, 2001; Moore et al., 2002; Kriegsfeld et al., 2004; Welsh et al., 2010).

Beyond comparisons between mRGC axons and nonretinal axons, the different features of nonretinal axons reveal much about the diversity of afferents innervating the SCN. We found two types of nonretinal boutons, those with or without intrusions of dendritic spines. These intrusions have been associated with several functions (driver vs modulator of neuronal activity; Sherman and Guillery, 1998). The more dendritic spine intrusions a bouton has the bigger this bouton is and the more synaptic partners and mitochondria this bouton would have. This could reflect that more mitochondria can support more synaptic activity and expand the volume of the bouton. The presence of these intrusions also suggests a difference in function and/or origin. We show here that almost no mRGC boutons have dendritic intrusions. We, therefore, hypothesize that axons originating from outside the SCN form small boutons that function as modulators, whereas axons of SCN neurons with boutons with dendritic intrusions work as drivers during intra-SCN communication. Previous anatomic studies of SCN synaptic afferents did not reveal dendritic intrusions in glutamatergic or serotoninergic axonic boutons (Kiss et al., 1984; Bosler and Beaudet, 1985; Castel et al., 1993). Intrusion-like features have been found in GABAergic boutons that do not express AVP, likely originating within the SCN (Castel and Morris, 2000), but no intrusions were observed after labeling VIP cells (Card et al., 1981). However, as the main objective of these studies was not to identify boutons with intrusions, it is difficult to conclude which types of boutons contain dendritic intrusions. Further studies are needed to clarify their origins and roles.

### Dendro-dendritic chemical synapses form a dense network in the SCN

Ultrastructural studies in the 1970s first identified dendro-dendritic chemical synapses in the SCN (Güldner and Wolff, 1974). They were found to be uncommon (Moore and Bernstein, 1989). Before that, this type of synapse was discovered between mitral and granule cells in the mammalian olfactory bulb, starting a discussion of the function of DDCS (Rall et al., 1966; Shepherd, 2009). Although DDCS in the olfactory system has been extensively studied, those in the SCN have been largely ignored.

In a previous study, we found that dendrites with DDCS form a network in the SCN core (Kim et al., 2019). Here, we confirmed the presence of a dense network of DDCS-positive dendrites in the core and found a less dense version in the shell. The limited volumes studied here prevented us from determining whether these two networks are interconnected or function in parallel, communicating through regular ADCS. The homogeneous synaptic input from mRGCs and nonretinal axons argues in favor of a continuous network. However, previous studies have shown that the core and the shell can be desynchronized during experiments such as jet lag (Albus et al., 2005; Nakamura et al., 2005; Davidson et al., 2009; Yamaguchi et al., 2013; Mei et al., 2018). In these conditions, the shell shows a rapid shift while the core shifts more slowly, suggesting separate processes for entrainment to the new light cycle.

Here, we noticed two types of DDCSs. Some have a very limited number of synaptic vesicles clustered at the synapse, whereas others have many synaptic vesicles. Unfortunately, the small volume analyzed prevented us from further characterizing these synapses. However, given that most of the core SCN neurons express GABA and VIP, we infer they are probably releasing GABA, VIP, or a combination. The precise role of DDCS in the SCN remains to be elucidated, but we hypothesize they play a role in communication between adjacent cells, as we failed to identify axo-dendritic or axo-somatic synapses from a proximal axon in either volume.

This type of communication may be crucial to synchronize SCN cells. Multiple lines of evidence indicate that the extracellular level of GABA is rhythmic (Hastings et al., 2018) and drives synchrony between SCN cells. This paracrine GABA could originate from DDCS rather than axonal synaptic terminals. This hypothesis fits with recent results showing a small-world synaptic network composed of interconnected nodes mainly in the core SCN. This network allows for rapid re-synchrony of neurons after tetrodotoxin treatment (Abel et al., 2016). In addition, because DDCS-positive dendrites receive more retinal and nonretinal input, these GABAergic synapses could modulate their responses in a phase-dependent manner. Further studies are needed to elucidate the precise role of this DDCS network in the SCN and its role in integrating synaptic input.

### SCN neurons contain specific intracellular organelles

In addition to the connectivity variation between regions of the SCN, this dataset allowed us to identify specific ultrastructures that cannot be observed otherwise. In particular, we were able to identify the presence of stigmoid bodies (SBs) in the mouse SCN, with a higher density in the shell compared with the core. SBs have been described as a structure morphologically similar to the nucleolus but located in the cytoplasm of neurons. While its exact function remains unclear, it is speculated to be involved in the aromatization of androgens to estrogens. This is because antibodies against the placental antigen associated with aromatase P-450 reveal that SBs are located in sex-steroid-sensitive peripheral tissues such as the ovaries and testes (Santolaya, 1973; Shinoda et al., 1993). Neurons containing SBs are present in multiple brain regions including the hypothalamus, thalamus, amygdala, septum, hippocampus, colliculi, and brainstem (Santolaya, 1973). It is worth noting that the differential distribution of stigmoid bodies in the SCN potentially reveals that the core and shell have different functions in the context of circadian rhythms. Multiple studies have found dot-like structures in the rodent brain that are 5-hydroxytrptamine_7_ (5-HT_7_) receptor immunoreactive (Muneoka and Takigawa, 2003). These structures match the size and location of the SBs we observed in the present study. The mouse SCN also expresses 5-HT_7_ receptors (Belenky and Pickard, 2001). Although linking SBs and serotonergic neurons could instigate an interesting discussion in the context of circadian regulation, this link is not thoroughly supported since EM analysis does not indicate that 5-HT_7_ localizes to SBs (Belenky and Pickard, 2001).

In summary, we established for the first time patterns of mRGC connectivity in two regions of the SCN, the core, and the shell, in a healthy mouse. We uncovered a multi-level system by which light regulates SCN activity. This work lays the foundation for studying the impact of neurodegenerative disease and circadian-related syndromes on neuronal connectivity.

### Limitations

One of the limitation of this study, similar to almost every SBEM studies, is that all data were obtained from a single animal and the imaged volume is only a fraction of the SCN. Our results, therefore, do not reflect potential interindividual and time-of-day variabilities. Nevertheless, many features characterized in detail here were in agreement with previous light microscopy or electron microscopy studies, thus supporting generalizability of our findings. Furthermore, the present study represents the largest and most detailed analysis of the SCN connectome. Importantly, we establish a solid foundation for more detailed analyses of the connectome that take into account the different parameters mentioned above. The rapidly evolving field of SBEM imaging, 3D reconstruction, and connectomics analysis can only benefit the results reported here.

## Synthesis

Reviewing Editor: Jibran Khokhar, University of Guelph Decisions are customarily a result of the Reviewing Editor and the peer reviewers coming together and discussing their recommendations until a consensus is reached. When revisions are invited, a fact-based synthesis statement explaining their decision and outlining what is needed to prepare a revision will be listed below. The following reviewer(s) agreed to reveal their identity: NONE. Note: If this manuscript was transferred from JNeurosci and a decision was made to accept the manuscript without peer review, a brief statement to this effect will instead be what is listed below.

The authors have done an excellent job of thoroughly addressing all the comments making this is a strong report for publication in eNeuro.
