# Supplementary figures and images for: Ultrastructure of Synaptic Connectivity within Subregions of the Suprachiasmatic Nucleus Revealed by a Genetically Encoded Tag and Serial Blockface Electron Microscopy
Source: eNeuro. 2023 Aug 23;10(8):ENEURO.0227-23.2023. doi: 10.1523/ENEURO.0227-23.2023 (PMC10449486; doi:10.1523/ENEURO.0227-23.2023)

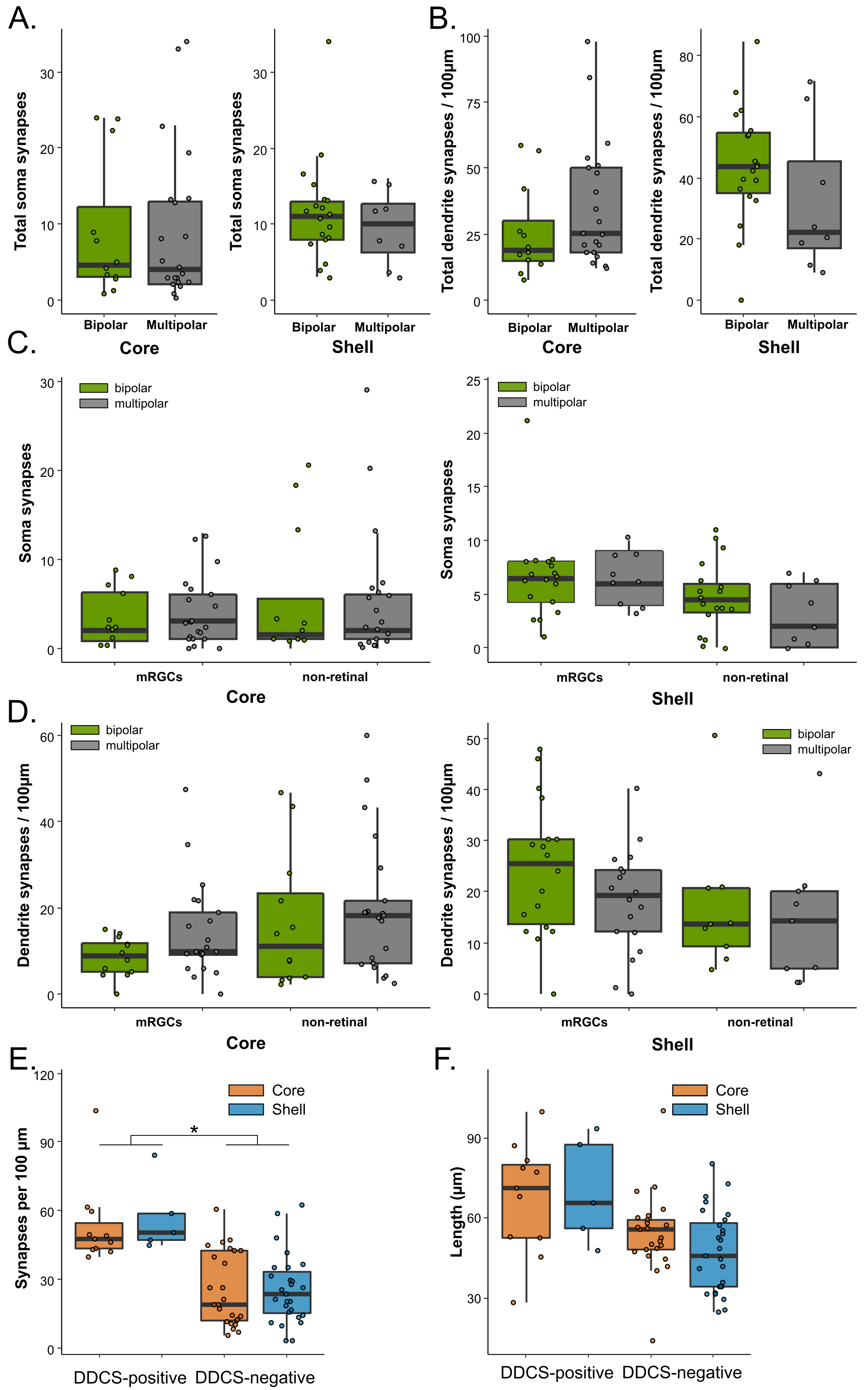

Supplement: Extended Data Figure 4-1 — Synaptic densities on soma, proximal dendrites and distal dendrites. A, B, Total synapse density on soma (A) and proximal dendrites (B) of bipolar and multipolar neurons in the core and the Shell. C, D, Density of mRGCs and nonretinal synapses on the soma (C) and proximal dendrites (D) of bipolar and multipolar neurons in the core and the shell. E, Linear density of ADCS on DDCS-positive and negative dendrites. F, Average length of DDCS-positive and negative dendrites; *p < 0.05. Download Figure 4-1, TIFF file. [file enu-eN-NWR-0227-23-s02.tiff]
